# Spatial sampling of terahertz fields with sub-wavelength accuracy via probe-beam encoding

**DOI:** 10.1038/s41377-019-0166-6

**Published:** 2019-06-12

**Authors:** Jiapeng Zhao, Yiwen E, Kaia Williams, Xi-Cheng Zhang, Robert W. Boyd

**Affiliations:** 1 The Institute of Optics, University of Rochester, Rochester, NY 14627 USA; 2 Department of Physics, University of Ottawa, Ottawa, ON K1N 6N5 Canada

**Keywords:** Imaging and sensing, Sub-wavelength optics, Terahertz optics

## Abstract

Recently, computational sampling methods have been implemented to spatially characterize terahertz (THz) fields. Previous methods usually rely on either specialized THz devices such as THz spatial light modulators or complicated systems requiring assistance from photon-excited free carriers with high-speed synchronization among multiple optical beams. Here, by spatially encoding an 800-nm near-infrared (NIR) probe beam through the use of an optical SLM, we demonstrate a simple sampling approach that can probe THz fields with a single-pixel camera. This design does not require any dedicated THz devices, semiconductors or nanofilms to modulate THz fields. Using computational algorithms, we successfully measure 128 × 128 field distributions with a 62-μm transverse spatial resolution, which is 15 times smaller than the central wavelength of the THz signal (940 μm). Benefitting from the non-invasive nature of THz radiation and sub-wavelength resolution of our system, this simple approach can be used in applications such as biomedical sensing, inspection of flaws in industrial products, and so on.

## Terahertz photonics: convenient characterization

A convenient and practical scheme for spatially characterizing terahertz (THz) signals has been invented by scientists in the US. The approach could prove useful for applications such as beam profiling, biomedical sensing, flaw detection and security screening. Jiapeng Zhao and coworkers from the University of Rochester make use of the electrooptic effect in a ZnTe crystal to indirectly measure the spatial profile of a THz signal with a resolution of *λ*/15 (62 microns), 128 × 128 sample points and kilohertz sampling speed. The approach relies on the fact that when a THz signal interacts with the ZnTe crystal it induces birefringence, which modifies the polarization of a near-infrared (NIR) probe beam. Spatially-modulating the NIR probe and measuring its polarization rotation via a single-pixel detector thus makes it possible to determine the profile of the THz beam.

## Introduction

The unique properties of terahertz (THz) radiation, such as its high transmittance through nonpolar materials and nonionizing photon energies, enable numerous novel possibilities in both fundamental research and industrial applications^1–7^. Consequently, the knowledge of the spatial profile of THz fields becomes very important. However, due to the lack of efficient and economical THz cameras^8,9^, characterizing the transverse structures of THz fields usually relies on raster scanning either the detector or the sample^10^, resulting in a low signal-to-noise ratio (SNR) and slow speed when the number of pixels increases. Recently, novel beam profiling approaches that involve computational sampling methods have emerged^11–17^. Computational sampling methods, which combine computational algorithms with optical imaging techniques, can improve the sampling speed and image quality, particularly under weak illumination^18^. These computational methods usually do not require cumbersome, conventional techniques such as multi-element THz detection arrays and raster scanning but can reconstruct the transverse fields with computational algorithms and a single-pixel camera. Nevertheless, much previous work has been based on spatially manipulating the THz beam directly with either dedicated THz spatial light modulators (SLMs)^11,12^, or modulating the THz spatial transmittance in semiconductors or nanofilms via photon-excited free carriers^13–17^. Compared to optical SLMs, the THz counterparts usually have a low temporal modulation rate and a large pixel size, which is mainly limited by the relatively long THz wavelengths, leading to a relatively low spatial modulation accuracy and spatial resolution^11,12^. Therefore, THz SLMs are not ideal devices for sampling applications that require sub-wavelength features. Although approaches that use spatial transmission modulation via photon-excited free carriers can provide precise spatial modulation on the scale of a few microns, these methods require one to fabricate samples onto semiconductors and use substantial laser power to excite free carriers. A high-speed synchronization among multiple optical beams is also necessary, which increases the level of system complexity^15^. Thus, it is important to develop a simpler THz spatial sampling approach that is capable of providing micron-scale accuracy for practical application scenarios.

The electrooptic (EO) effect is one of the most widely used THz techniques for coherent detection^19^. When a THz field interacts with an EO crystal (usually ZnTe or GaP), it introduces birefringence, which modifies the polarization of a co-propagating 800-nm near-infrared (NIR) probe beam. This rotation in polarization is measured to determine the time-dependent THz electric field. Therefore, only THz fields that spatiotemporally overlap with the NIR probe beam result in a measurable polarization shift. From a sampling point of view, when we encode the NIR beam with desired patterns and carefully align these patterns with THz radiation, the fields of interest can be selectively measured. Since we only spatially modulate the NIR probe beam, this indirect measurement is not only much easier than manipulating the THz fields directly but also preserves the spatial information of the THz field as well. The non-destructive nature of this method may also enable quantum measurements of THz fields in the future. Furthermore, considering that the spatial manipulation is realized using comparatively economical and well-developed optical SLMs, achieving fast temporal modulation rates and sampling accuracies of a few microns is also feasible^20^. Therefore, real-time sensing through the use of our probe-beam-encoding technique should be achievable in the future.

In the work reported in this paper, we determine the spatial distribution of THz fields by encoding an 800-nm NIR probe beam using a commercially available NIR SLM. This device provides a fast sampling rate up to the kilohertz (kHz) level and a sampling accuracy up to a few microns. Note that, in this work, the sampling accuracy refers to the size of the minimum sampling size that the NIR probe beam can provide, which corresponds to the pixel size of the recovered image. As a demonstration of our method, we determine the spatial profile of an object surrounded by a material (paper) that is opaque to NIR and visible radiation but transparent to THz radiation. We accomplish this task by impressing a series of spatial masks onto a NIR probe beam and simultaneously pass a THz beam through the object. Through the use of the EO effect, the spatial information of the THz beam is transferred to the NIR probe beam, whose total power is then measured. After repeating this procedure for each spatial mask, the THz field distribution carrying near-field information is retrieved with 128 × 128 sample points through the use of the Hadamard Matrix (HM) algorithm^21^. The spatial resolution, which is defined as the size of the smallest resolvable feature, is estimated to be 62 μm. This resolution is 15 times smaller than the THz central wavelength (940 μm at 0.32 THz). By adopting the compressed sensing (CS) algorithm, we can recover high-fidelity fields (over 95% fidelity) with a sampling ratio of 50%. This simple technique provides micron-scale sampling accuracy and a sub-wavelength resolution while inheriting most of the advantages of THz sensing, such as broadband spectrum information and non-invasive examination of biomedical samples.

## Results

### Experimental procedure

**Fig. 1:**
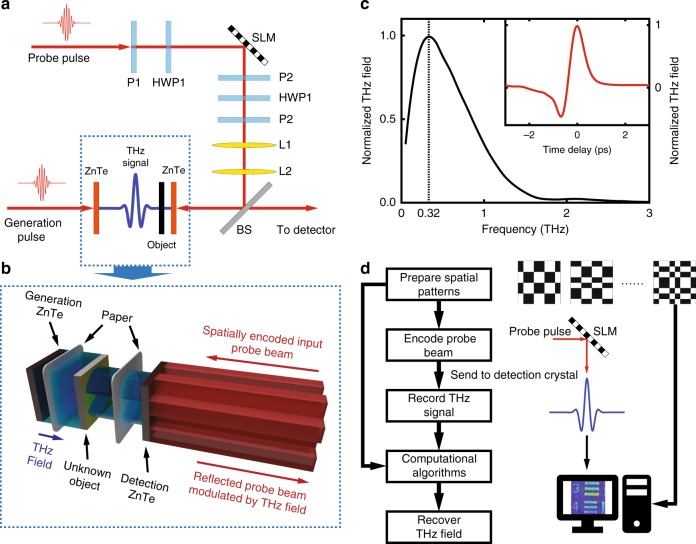
Experimental Configuration. **a** Schematic of the system configuration. **b** A detailed description of our sampling approach. The unknown object, an AF test chart, is wrapped in paper so that it is invisible to the NIR probe beam. P1, P2, and P3 are polarizers, while HWP1 and HWP2 represent half-wave plates. L1 and L2 image the SLM plane onto the left surface of the ZnTe detection crystal. BS is a 50/50 beam splitter. The probe beam, whose polarization has already been modulated, is then sent to a single-pixel detector for measurement. Detailed specifications can be found in the Methods section. **c** The spectrum of the generated THz pulse, and the time-domain electric field distribution with a spatial mask encoded on the NIR probe beam. The spectral peak occurs at 0.32 THz, which corresponds to a central wavelength of 940 μm. The SNR of the THz signal is ~300. **d** The flowchart shows our procedure for single-pixel imaging via probe-beam encoding

A THz pulse, generated through optical rectification from a ZnTe crystal, as shown in the schematic in Fig. 1a, b, passes through a covered object and is detected through EO sampling by another ZnTe crystal^22^. A sequence of spatial masks is loaded onto the SLM to encode the NIR probe pulse. This spatially encoded 800-nm probe beam first illuminates the ZnTe detection crystal in the counter-propagation direction of the THz pulse, as shown in Fig. 1b. Following reflection from both surfaces of the ZnTe detection crystal, two reflected NIR beams co-propagate with the THz pulse. However, only the polarization of the beam reflected from the left surface of the crystal is modulated by the spatiotemporally coincident THz field. To separate these two reflected beams, the ZnTe detection crystal is slightly tilted, and one lens is then used to focus the reflected beams (not shown in the figure). With the help of an iris at the focus, the beam carrying information is selected and sent to the single-pixel detector, which consists of a quarter-wave plate, Wollaston prism and balanced photodiode detector, to retrieve the time-dependent THz signal. The original time-domain THz pulse and corresponding spectrum are shown in Fig. 1c, respectively. The object, a positive US Air Force (AF) target (positive entails that all the strips are opaque on a transparent substrate) made with chromium, is wrapped in a 70-μm-thick piece of paper and placed immediately before the detection crystal. Therefore, the THz pulse only travels ~70 μm before interacting with the detection crystal, and the near-field information is maintained^23^. As shown in the flowchart in Fig. 1d, after recording the THz signal and the corresponding patterns, the THz transverse field distributions can be reconstructed with computational algorithms. Please note that the measurement point is held at the peak of the THz field during the imaging process.

### Resolution limit

**Fig. 2:**
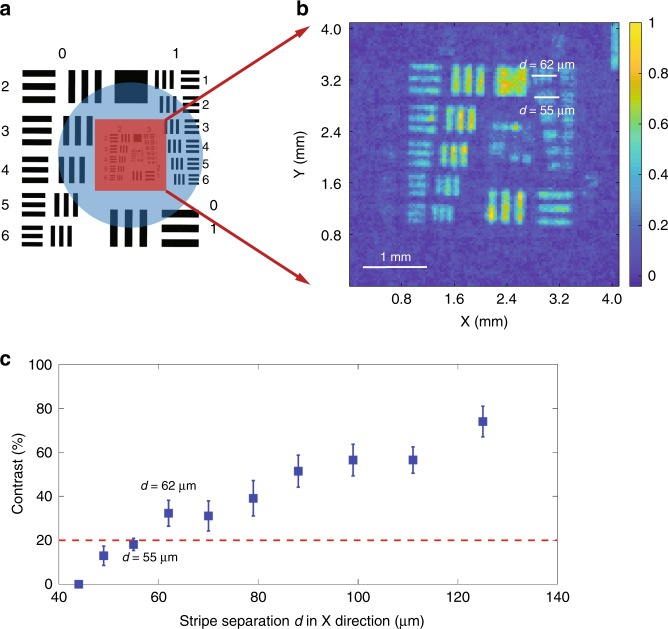
Resolution Estimation. **a** The original AF target. The red square indicates the area that the probe beam illuminates. The blue circle shows the parts illuminated by the THz field. By changing the location of the encoding masks on the SLM, we can probe any part of the THz field in the blue circle without changing the optics. **b** The experimental result with 128 × 128 pixels showing the resolution limit, which is the recovered image of the red square part in **a**. **c** The contrast as a function of strip separation *d*. The red dashed line is the threshold contrast assumed by the Rayleigh criterion. Two points can be resolved if the contrast is equal to or >20%. Therefore, the element set with *d* = 55 μm is not resolved while the element set with *d* = 62 μm is resolved according to the Rayleigh criterion. The intensity cross sections of element sets 3–1 and 3–2 can be found in the Fig. S1 in the Supplementary Information

We first use the HM algorithm to probe the THz field and successfully recover the field distribution with 128 × 128 sample points. As shown in Fig. 2a, we selectively sample the central elements of the AF chart to estimate the spatial resolution limit. The recovered intensity distribution is shown in Fig. 2b, which has a square sampling pixel with a width of 32 μm. In Fig. 2c, we show the contrast of each element in group 2 as well as element 3–1 (the first set of elements in group 3) and 3–2 as a function of strip separation *d* in the X direction. We find that the element with *d* = 55 μm (element 3–2) has an average contrast of 18.10%, while the element with *d* = 62 μm (element 3–1) has an average contrast of 32.28%. According to the Rayleigh criterion, two points are barely resolved with a contrast equal to 20% (red dashed line in Fig. 2c); the resolution of our system is found to be 62 μm. Considering that the central THz wavelength (*λ*) is 940 μm, we have achieved a resolution of ~*λ*/15. It should be noted that the features in the X direction are better resolved than those in the Y direction due to the horizontal polarization of the THz beam^15^. A better resolution in the Y direction can be expected when the THz field is vertically polarized. To obtain a better resolution for features in all directions, a circularly polarized THz field may be used. Since the strip separations in elements 3–1 and 3–2 are 62 μm and 55 μm, respectively, but the sampling pixel is 32 μm, we can see strong pixelization effects in the experimental data. The pixelization blurs the reconstructed image and further limits the resolution of our scheme. Through the simulations shown in the 4th section of the Supplementary Information, we find that the resolution in our configuration (with a 32-μm pixel size) is less than 35 μm. This resolution limit can be further improved to 11 μm (*λ*/85.5) if the pixel size is 8 μm. Another interesting observation from the simulation shows that a longer central wavelength can provide a better spatial resolution. This counter-intuitive conclusion arises from the nature of near-field imaging. As analyzed in previous work^23^, for the diffraction field of a sub-wavelength object in the near-field region, a small ratio of *z*/*λ* can maintain more features, where *z* is the propagation distance. Therefore, the factors that limit the spatial resolution include the pixel size, thickness of the detection crystal, central wavelength of the THz pulse, and the separation between the detection crystal and sample. Thus, we can further improve the resolution to a few microns by using a longer wavelength THz pulse, a thinner crystal with a larger nonlinearity, by encoding the probe beam with a smaller pixel size, or by moving the sample closer to the detection crystal^24,25^.

Note that our scheme can probe any portion of the THz field by solely changing the location of the NIR probe on the THz field through relocation of the encoding masks on the SLM. For instance, the results that we show above use the central part of the SLM for encoding, which yields a reconstructed field of the central part of the THz field.

### Comparison of different algorithms

In traditional THz beam profiling, raster scanning is the prevalent single-pixel sampling technique due to the lack of economical high-performance cameras. The limitations in speed and contrast become apparent when the total number of sampling points increases. With a finer sampling and an increased number of pixels, the SNR on each pixel is reduced due to the reduction in the signal level on each pixel. As a result, one needs to significantly increase the integration time in order to average out the noise. Furthermore, finer sampling also highly relies on precise mechanical controls. To overcome these limitations, computational techniques that project multi-pixel spatial masks, including random patterns and HM are introduced that remove the requirement of mechanical scanning and result in an accurate sampling control. As multiple pixels are sampled in each measurement, the limitations due to weak illumination are mitigated. Moreover, different algorithms can provide various benefits with respect to image quality and reconstruction speed. For example, when the source noise is negligible, the HM algorithm that minimizes the mean squared error gives the best SNR^21^, and the CS algorithm can be used to reconstruct the field with sub-Nyquist sampling rates for a fast measurement (see the 2nd section in the Supplementary Information)^26^.

**Fig. 3:**
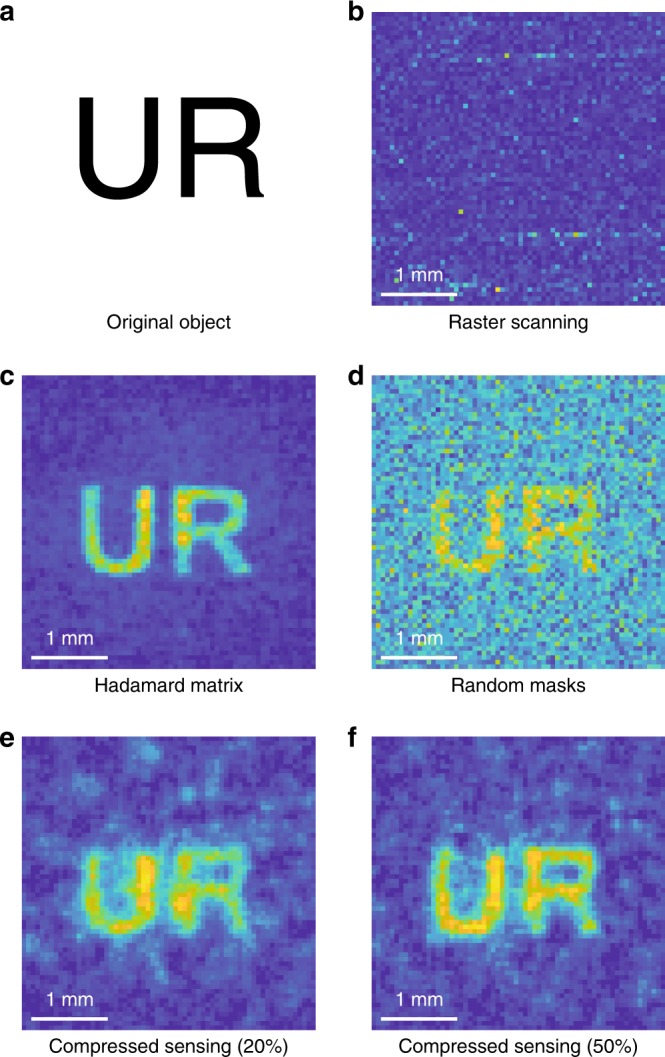
Algorithm Comparison. **a** Original ‘UR’ object. **b** Field distribution obtained by raster scanning. **c** Field distribution reconstructed by the HM algorithm. The bright central part of the ‘UR’ arises from the non-uniformly distributed THz field. **d** Reconstructed field obtained by random masks. **e** and **f** Recovered field obtained through the use of CS with different sampling ratios. All recovered fields have 64 × 64 sampling points with a pixel size of 64 μm

The experimental comparison of raster scanning, HM and CS algorithms is shown in Fig. 3. The object is a positive ‘UR’ mask (Fig. 3a) with a 300-μm linewidth (*λ*/3.13). In concept, the raster scanning entails illuminating each pixel sequentially and recording the THz signal of the corresponding pixel. This approach will not reveal any information of the field if the signal on each scanning pixel is smaller than the detector noise. Therefore, to make this comparison more convincing, we intentionally increase the pixel size from 32 μm to 64 μm to give an advantage to raster scanning. In this case, the incident power on each pixel is four times larger than the recovered field shown in Fig. 2b, even though the intensity is the same. However in this scenario, by comparing the recovered normalized field distributions obtained from raster scanning (Fig. 3b), HM (Fig. 3c), random binary masks (Fig. 3d) and CS (Fig. 3e, f), we can still see that all the computational algorithms have a better SNR than raster scanning under the same acquisition time. This is because for the pixel size of 64 μm, which is much smaller than the wavelength, the signal of the THz field on each single pixel is still smaller than the detector noise. Therefore, the results measured by raster scanning only give the detector noise but do not reveal any spatial information about the THz field. By comparing the results from all computational algorithms, we can see that the HM algorithm provides the best contrast, while the image reconstructed from random masks is the noisiest, as expected. By sub-sampling the field with a 20% sampling ratio, the CS yields a field distribution with 89.6% fidelity. Fidelity is defined as the correlation coefficients between the recovered THz field and the original object. With a 50% sampling ratio, we can achieve 96.9% fidelity, which is mainly limited by the background noise. One can further improve the fidelity by adding more image processing algorithms in the restoration stage, which is beyond the scope of this paper. Therefore, we believe that the field recovered by the CS algorithm with a 50% sampling ratio is the best choice for real applications. This conclusion derives from the fact that high-fidelity field distributions are accessible with only half of the original total acquisition time (compared to the HM algorithm) by sacrificing a tolerable amount of information. A higher fidelity may also be available if one combines our technique with more advanced imaging processing algorithms.

A faster acquisition time is mainly limited by the low switching speed of the SLM (60 Hz) and will be further limited by the repetition rate of our laser (1 kHz) if high-speed (kHz-level) digital micromirror devices (DMDs) are used as the new SLM. Since a THz field with a uniform spatial distribution is the only requirement for a high-quality field reconstruction, we can avoid using high pulse energy amplifier lasers with low repetition rates. Therefore, a spintronic THz emitter pumped by a high repetition rate oscillator laser can be employed for ultrafast sampling^27^, which can possibly lead to real-time beam profiling and future integrated THz imaging devices^20^. Note that, to obtain an accurate estimation of the resolution limit, our demonstration focuses on binary sparse objects. However, these computational imaging methods can work well for non-sparse and non-binary objects, which means that these approaches are practical for real applications^20,28,29^.

## Discussion

We have demonstrated that our near-field spatial sampling technique can provide sub-wavelength resolution with high-fidelity through the use of a spatially encoded probe, and have shown the possibility of improving this technique to achieve real-time THz imaging with both amplitude and spectral information^30,31^. In comparison to raster scanning methods, our approach provides a better sampling accuracy, resolution, and contrast. These advantages are facilitated by computational algorithms that offer a general advantage over all THz imaging methods based on raster scanning^2,18^.

In relation to EO imaging^25,32^, our approach does not measure the THz spatial profile directly but recovers the transverse field distribution through the use of computational algorithms. This non-destructive measurement gives rise to better performance in terms of both resolution and contrast without requiring high-power lasers, especially when the detection crystal is thick^33^. As shown in the 4th section in the Supplementary Information, our approach can provide a resolution of 9 μm (*λ*/107) when the detection crystal is 20 μm thick and the separation between the crystal and object is 70 μm. This resolution is 1.4 times better than the resolution achieved in a conventional EO imaging system with the same crystal but a separation of 3 μm^25^. Additionally, indirectly sampling the THz field can circumvent the requirements of using high-energy lasers, which is a common requirement of most THz sub-wavelength imaging techniques involving not only traditional EO imaging but also techniques based on laser filamentation^34^.

Regarding computational imaging approaches with dedicated THz SLMs, the advantages in our method mainly arise from the use of optical SLMs. As we showed earlier, THz SLMs usually have a lower modulation rate, which renders them impractical for real applications. Their large pixel size also limits the resolution to a few hundreds of microns even in the near-field region^8,9^. Lastly, THz SLMs tend to be custom designed and fabricated, which renders them too expensive for many applications.

Compared to imaging techniques based on the photon-excitation of free carriers, probe-beam encoding eliminates the reliance on complicated high-speed synchronization among three arms, which makes the system both optically and electronically less complicated^12–14^. Another advantage is that there is no need to fabricate samples onto semiconductor substrates, which is more convenient for most applications such as biomedical imaging. As we have shown in Fig. 3, samples can be put directly onto a coverslip for imaging, which is thus as simple as using a conventional microscope in biomedical sensing.

In comparison with the novel THz nonlinear ghost imaging technique^35^, our approach can provide a better resolution and SNR. Through encoding the spatial pattern directly onto the pump beam, the THz nonlinear ghost imaging technique can conveniently generate a structured THz field to achieve computational imaging. However, this also limits the thickness of the generation crystal. As shown in refs. ^23,33^, sub-wavelength structures will become rapidly blurred upon propagation. Therefore, to obtain an accurate sampling, the thickness of the generation crystal has to be limited to a thin crystal, which will lead to a weak THz field and, hence, a low-SNR recovered image. Otherwise, if a thick generation crystal is used, the spatial resolution will be limited to a lower level.

In summary, we demonstrate a concise and robust method to spatially sample THz fields up to kHz level sampling rates and a sampling accuracy of a few microns. With this approach, we demonstrate a THz near-field sampling system using a single-pixel THz detector, and use this system to successfully measure an object with 62-μm (*λ*/15) resolution. By adopting the CS algorithm, we can recover high-fidelity field distributions (near 95% fidelity) by sub-sampling the THz beam, providing a way to achieve fast beam profiling. We believe that such a tool can provide numerous future applications, including lossless beam profiling, biomedical sensing, flaw detection, and security inspection.

## Materials and methods

### Experimental parameters

An 800-nm Ti:sapphire amplifier laser (Coherent Legend Elite Duo with seed laser Coherent Vitara S) with a 1-kHz repetition rate is used. The pulse duration was measured to be 100 fs by an autocorrelator. The collimated beam is split by a 90/10 beam splitter (BS1) at the front of the set-up. After the delay line, the pump beam illuminates a 10 × 10 × 1 mm ZnTe crystal to generate the THz pulse using optical rectification^22^. A 10-mm-diameter iris is used before the generation crystal to select the center part of the beam, which gives a relatively uniform intensity distribution for the pump beam. A power density of ~1.27 W/cm^2^ is used to generate THz radiation, corresponding to 1 mJ of pulse energy. The generated THz beam illuminates an ‘unknown’ object, which is wrapped in a 70-μm-thick paper sheet after traveling through a silicon wafer that blocks the residual 800-nm pump beam. The paper is opaque to 800 nm and visible light. The NIR probe beam travels through a 15-mm-diameter iris and is imaged onto the SLM screen (Hamamatsu LCOS-SLM X10468-02 with a pixel size of 20 μm). The imaging system consists of one 25-cm focal-length lens and one 20-cm focal-length lens (not shown in the figure), so that the magnification is 0.8. The beam diameter on the SLM is 12 mm. The SLM spatially encodes phase-only patterns onto the probe arm, and we then use a common-path interferometer to transfer these phase-only patterns to intensity patterns. The common-path interferometer consists of two polarizers (P1 and P2) and the HWP1. P1 is located before the SLM to make sure that the polarization is horizontal. The half-wave plate 1 (HWP1) then rotates the polarization to 45^◦^. P2 is also set to 45^◦^ and located after the SLM. Another HWP2 is used after P2 to rotate the polarization back to the horizontal direction. In the experiment, we observe that when we switch the patterns on the SLM, the polarization state of the beam is also slightly changed, which leads to noise on the balanced detector. Therefore, two Glan-Taylor polarizers (GCL-0702) (P3) are used to increase the extinction ratio in the horizontal direction so that all the vertical polarization gets rejected after P3. A 25-cm focal-length lens (L1) and a 20-cm focal-length lens (L2) form an imaging system to image the SLM plane onto the left surface of the ZnTe detection crystal. This leads to a 16 μm SLM pixel size on the detection crystal plane, which indicates that our sampling pixel either consists of 2 × 2 pixels on the SLM (for images with 32 μm pixel size) or 4 × 4 pixels on the SLM (for images with 64 μm pixel size). On reflection from a 50/50 beam splitter (BS), the probe beam carrying spatial patterns propagates to the ZnTe detection crystal. The translation stage is well aligned to make sure that the probe pattern spatially overlaps with the THz field of interest and temporally overlaps with the peak position of the THz pulse. As we have shown in the main text, we intentionally select an NIR probe beam, which is reflected from the left surface of the crystal by using a lens (*f* = 25 cm) and an iris. Then, this NIR probe beam, which carries information, is collimated and sent to a quarter-wave plate followed by a Wollaston prism. Another lens is used to focus the probe beam onto the balanced detector. The intensity in the probe beam is ~0.1 mW/cm^2^, corresponding to a pulse energy of 16.8 nJ.

The unknown object used to identify the resolution limit is a positive US Air Force target, which matches MIL-S-150A standard (Thorlabs R3L3S1P). The target is made from 120-nm-thick chrome deposited onto a 1.5-mm-thick clear soda lime glass substrate. The surface with the test strips faces the detection crystal, so that the THz field carrying the object information travels a distance of only 70 μm before interacting with the ZnTe detection crystal, whose dimensions are 10 × 10 × 0.1 mm. Unlike a conventional THz measurement scheme, our approach utilizes the entire surface of the detection crystal, which means that any non-uniformity in the nonlinearity (i.e., *r*_14_(x,y) as a function of the transverse coordinates) may also lead to a decrease in image quality. More details can be found in the 1st section of the Supplementary Information.

### Measurement parameters

To obtain a fair comparison and explicitly show the advantage of computational imaging, the intensity of the THz illumination and the NIR probe beam as well as the total acquisition times for all reconstructed fields shown in Fig. 3 are set to be the same (except for the fields recovered using compressed sensing). As a result, the raster scanning we used is not a typical technique, which involves a focused THz radiation and a focused NIR probe beam with a two-dimensional scanning stage. The integration time of the detector is 0.03 s. The total measurement time for each spatial pattern is 0.5 s, while the total measurement time for each scan for raster scanning is 1 s. Therefore, the total acquisition time for a recovered field with 128 × 128 sample points is ~5 h, which is roughly three times faster than that of previous work^13^. The scanning range at the image plane is 4.096 × 4.096 mm^2^. The details of how we probe the field using different algorithms can be found in the 1st section of the Supplementary Information. Here, we need to emphasize that the brighter center part and the relatively dimmer outside of the recovered fields are caused by the Gaussian spatial distribution of the THz field and the NIR probe beam, which both have stronger intensity at the central part. One possible solution to this is the use of telescopes with large magnifications to expand both beams.

The ‘UR’ sample is positively fabricated with 100-nm-thick chromium on a 170-μm-thick coverslip via physical vapor deposition (PVD). The sample is wrapped in paper with the characters facing the ZnTe detection crystal, which results in a separation distance of ~70 μm between the sample and detection crystal. All the experiments were carried out at room temperature, so this technique should directly fit in with most applicable scenarios.

## Supplementary information

Supplementary Information
