# Supplementary material for: Spatial sampling of terahertz fields with sub-wavelength accuracy via probe-beam encoding
Source: Light Sci Appl. 2019 Jun 12;8:55. doi: 10.1038/s41377-019-0166-6 (PMC6559961; doi:10.1038/s41377-019-0166-6)
Supplement: Supplementary file 1 — Supplementary Information [file 41377_2019_166_MOESM1_ESM.pdf]

# Supplementary Information
# for
# Spatial Sampling of Terahertz Fields with Sub-wavelength Accuracy via Probe-Beam Encoding

Jiapeng Zhao[1], Yiwen E[1], Kaia Williams[1], Xi-Cheng Zhang[1, *], and Robert W. Boyd[1, 2, *]

1. The Institute of Optics, University of Rochester, Rochester, New York, USA, 14627

2. Department of Physics, University of Ottawa, Ottawa, Canada, K1N 6N5

Corresponding authors:

Xi-cheng Zhang (xi-cheng.zhang@rochester.edu),

Robert W. Boyd (boydrw@mac.com).

## I. THEORY OF THZ SINGLE-PIXEL IMAGING USING ELECTRO-OPTIC EFFECT

The electro-optic (EO) effect can be used to accurately measure the amplitude and phase of THz fields [1, 2]. The THz pulses introduce birefringence in EO crystals which changes the polarization of the probe beam. By using the quarter wave plate (QWP) and Wollaston prism, the probe beam will be separated into two beams with equal intensity but orthogonal polarization if there is no overlap with the THz beam. After the THz beam modulates the polarization of the probe beam, the signal $P_s(t)$ on the balance detector has the following relation with the THz field $E_{THz}(x, y, \omega_{THz}, t)$ [1]:

$$P_s(t) = \int\int \frac{1}{c} I_0(x, y, \omega_1)\omega_1 L n_0^3(\omega_1) r_{14}(x, y) E_{THz}(x, y, \omega_{THz}, t) dxdy, \quad (1)$$

where $c$ is the speed of light in vacuum, $I_0(x, y, \omega_1)$ is the intensity distribution of the probe on the detection crystal. $L$ is the thickness of the detection crystal, and $\omega_1$ is the frequency of probe. $n_0(\omega_1)$ is the refractive index of the detection crystal at optical frequency $\omega_1$, and $r_{14}(x, y)$ is the EO efficient of the detection crystal. From Eqn. (1), one can see that the THz field can only be detected if it both spatially and temporally coincides with the probe on the detection crystal. Therefore, solely manipulating the probe arm in the spatial domain is equivalent to spatially modulating the THz field in the same manner but leaving the probe field unchanged. Moreover, from the measurement point of view, as long as the polarization of the optical probe is kept, spatially changing the probe field will not change the signal on the balanced detector. Therefore, in the THz computational sampling, it does not matter whether we manipulate the THz field or the probe field. However, even both methods give the same result in principle, encoding patterns on the probe beam can give more benefits as what we discuss in the main paper. Therefore, we can use probe beam encoding to combine computational imaging algorithms and THz imaging techniques.

Recently, computational imaging becomes a promising candidate of single-pixel imaging methods. In essence, measuring a $N$ pixel field, i.e. imaging a field, is equivalent to measure an unknown vector $\psi$ in a N dimensional Hilbert space. Therefore, this unknown vector can be decomposed into a set of complete orthogonal basis $H$ in that N dimensional Hilbert space with a coefficient set $\phi$. We can rewrite this decomposition into the matrix form: $\psi = H\phi$, where $\psi$ and $\phi$ are two $N$ dimensional column vectors representing the unknown vector and the coefficients of each basis vector respectively. $H$ is a $N \times N$ matrix and each row represents a basis vector in the Hilbert space. Therefore, one can reconstruct the unknown vector $\psi$ by measuring the coefficients sets $\phi$ in the

complete orthogonal basis $H$, and these coefficients can be found as $\phi = H^{-1}\psi$. In our case, this complete orthogonal basis is Hadamard Matrix (HM), which can minimize the mean square error of the image [3]. HM is a square matrix with $+1$ or $-1$ elements, and the rows of HM are mutually orthogonal. Therefore it satisfies the relation: $H_N H_N^T = H_N^T H_N = N I_N$, where $H_N$ is the HM in a $N$ dimensional Hilbert space, $H_N^T$ is the transpose of $H_N$ and $I_N$ is the $N$ dimensional identity matrix. The HM consists of $+1$ or $-1$ elements, but our SLM can only provide $+1$ or $0$ elements for on and off measurements. Thus, we decompose the HM matrix into $H_{N,1}$ and $H_{N,-1}$ which carries only $+1$ or $0$ elements and $-1$ or $0$ elements respectively: $H_N = H_{N,1} + H_{N,-1} = H_{N,1} - |H_{N,-1}|$. Considering the rules of linear algebra, we have the following relation to reconstruct the image:

$$\psi = (H_{N,1} - |H_{N,-1}|)\phi, \tag{2}$$

where both $H_{N,1}$ and $|H_{N,-1}|$ only carry $+1$ or $0$ elements so that they can be generated from SLM directly. Therefore, in the experiment, we encode the probe beam with $H_{N,1}$ and $|H_{N,-1}|$ patterns sequentially, and record the corresponding signals. To reconstruct the image, one needs to subtract the $|H_{N,-1}|$ patterns weighted by the corresponding signal from $H_{N,1}$ patterns weighted by the corresponding signal. Although this differential method will double the measurement time, it can eliminate the source noise if one immediately shines the $|H_{N,-1}|$ mask right after the corresponding $H_{N,1}$ mask. That is to say, one does not need to introduce any additional power monitor to track the laser power fluctuation.

Now we consider an unknown object with intensity distribution $O(x, y)$ placed before the detection crystal. For a given $i_{\text{th}}$ HM pattern $I_i(x, y, \omega_1)$ which is imaged from SLM plane onto the detection ZnTe crystal with thickness $d = z_1 - z_0$, we can find the measured THz total field $P_{\text{s},i}$ from Eqn. (1) as:

$$P_{\text{s},i}(t) = \int\int\int_{z_0}^{z_1} \frac{1}{c} I_i(x, y, z, \omega_1)\omega_1 L n_0(\omega_1)^3 r_{14}(x, y) E_{\text{THz}}(x, y, z, \omega_{\text{THz}}, t)\, dx dy dz, \tag{3}$$

where $E_{\text{THz}}(x, y, z, \omega_{\text{THz}}, t)$ can be found from scalar diffraction theory using angular spectrum:

$$E_{\text{THz}}(x, y, z, \omega_{\text{THz}}, t) = \mathcal{F}^{-1}\{\mathcal{F}[O(x, y, 0)] exp[i2\pi\sqrt{(k_{\text{THz}}/2\pi)^2 - f_x^2 - f_y^2} z]\}. \tag{4}$$

The $\mathcal{F}$ and $\mathcal{F}^{-1}$ represent Fourier Transform and Inverse Fourier Transform respectively. $f_x$ and $f_y$ are spatial frequencies in $x$ and $y$ directions. Here, we have assumed that the THz wave has a uniformly distributed intensity. One can then find the corresponding HM pattern $I_i(x, y, z, \omega_1)$ using Eqn. (4). With $I_i(x, y, z, \omega_1)$ and $E_{\text{THz}}(x, y, z, \omega_{\text{THz}}, t)$, we can find the signal $P_{\text{s},i}$ for the

$i_{th}$ pattern. By repeating this procedure for all patterns, we can get the signal set $P_{s,i}$ which form the coefficient vector $\phi$. This coefficient vector will be used to reconstruct the image vector $\psi$ through Eqn. (2). To show the state of art, the analysis here does not include the broadband nature of THz pulse. However, the simulation we will show already take the spectra contribution into consideration.

## II. THEORY OF COMPRESSED SENSING

The basic idea of compressed sensing comes from the fact that, for a $N$ dimensional vector $\psi$, it is possible to find an orthogonal basis $T$ that most coefficients in the coefficient set $\phi$ are zero or very small. Due to this sparsity in the vector decomposition, there will no much information loss if we discard these small or zero coefficients. Therefore, one can recover the original vector $\psi$ with a high fidelity using only those large coefficients. Now we assume the THz field can still be represented by a $N$ dimensional column vector $\psi$ in the Hilbert space. In the conventional linear solutions, as what we discuss in the last section, we can write the solution to $\psi$ in the matrix form as: $\phi = Q\psi$, where $Q$ represents the linear transformation matrix representing the basis, and $\phi$ still represents the coefficients set. Now we consider another set of basis represented by $T$, which is assumed to be incoherent with basis $Q$. If we assume that there are only few nonzero coefficients under this transformation $T$, we can recover the $\psi$ using a nonlinear strategy by solving the convex optimization problem with [4]:

$$\min_{\psi'} \|T\psi'\|_{\ell_1}, \text{subject to } Q\psi' = \phi, \tag{5}$$

where $\|\cdot\|_{\ell_1}$ is the 1-norm. If the coherent coefficient between two bases $Q$ and $T$ is very little, i.e. two bases are incoherent, the THz field vector $\psi$ can be reconstructed with $M \geq O[Klog(N)]$ measurements, where $K$ is the number of nonzero components of vector $T\psi$ [5]. Therefore, we can sub-sample the THz field by the Nyquist-Shannon law but still recover the field with a high fidelity.

From the practical point of view, we use the TVAL3 package to solve the minimization problem. The package was provided by Chengbo Li, Wotao Yin and Yin Zhang from Rice University [6]. There are 4 different models available and we use TV/L2+ model for our field reconstruction. More than 10 parameters can be adjusted in the model. In principle, it is possible to improve the fidelity by carefully adjusting each parameter. However, this will be trivial and beyond the scope of our

work.

## III. COMMON PATH INTERFEROMETER

If we illuminate an arbitrarily polarized light onto the liquid crystal SLM, only the horizontally polarized portion gets modulated. Therefore, through the utilization of this property, we will use common path interferometer to transfer the phase-only spatial patterns into intensity-only patterns. In our case, the axis of HWP 1 in Fig. 1(a) is fixed at 22.5° so that the polarization of the optical probe beam is changed from horizontal polarization to diagonal polarization (45° to the horizontal direction). We can write the polarization state $|D\rangle$ before the SLM as:

$$|D\rangle = \frac{1}{\sqrt{2}}(|H\rangle + |V\rangle), \tag{6}$$

where $|H\rangle$ and $|V\rangle$ represent the horizontal and vertical polarization states respectively. The phase masks on the SLM are binary masks with 0 and $\pi$ phase delay. Therefore, after reflected by the SLM, the polarization of areas with $\pi$ phase delay is in anti-diagonal polarization state $|A\rangle$. If we assume the binary spatial phase mask on the SLM can be represented by term $\psi(x, y)$, we will find the state after the SLM $|D\rangle_{\text{SLM}}$ in the form:

$$|D\rangle_{\text{SLM}} = \frac{1}{\sqrt{2}}(|H\rangle + exp(i\psi(x, y))|V\rangle). \tag{7}$$

Right after the SLM, we use a polarizer with its axis at 45° to interfere the $|H\rangle$ and $|V\rangle$ components:

$$\langle D|D\rangle_{\text{SLM}} = \frac{1}{2}(1 + exp(i\psi(x, y))). \tag{8}$$

For those parts with the phase delay equal to $\pi$, Eqn. (4) will become 0, and the 0 phase delay parts will become unity. Hence, we get a binary intensity pattern from the phase-only SLM using common path interferometer.

Even though the intensity-only device such as digital micromirror device (DMD) has a much higher speed (kHz level), there are two advantages of using the phase-only SLM. Since the micromirror array, which basically works like a grating, will lead to strong diffraction to the incoming beam, these DMDs usually have low efficiencies. The other drawback is that, due to the reflection nature of micromirror array, it will spatially shear the pulse front. In our test, the duration of a pulse can be stretched from 200 $fs$ to 4 $ps$. Therefore, in order to use the DMD, one has to compensate this spatial shear effect. A recently published work also discussing this effect and applying it to THz pulse generation when we prepare the draft [7].

## IV. RESOLUTION ESTIMATION

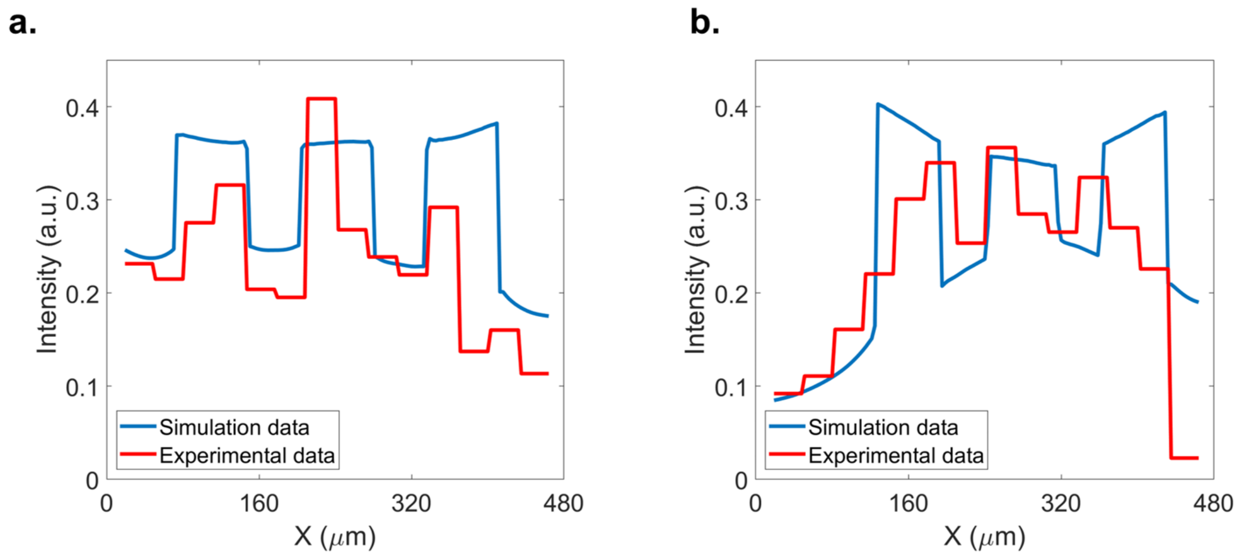


Fig. S1: (a) and (b) are intensity cross sections of element set 3-1 and 3-2 in Fig. 2(b) in the main text respectively.

We first define the spatial resolution as the size of the smallest resolvable feature in the image of the object. The criterion to justify whether the feature can be âĂŹresolvedâĂŹ is Rayleigh criterion, which yields a 20% contrast ratio if the feature is barely resolved. Therefore, in our resolution estimation procedure, we first take the intensity cross sections of different element sets and then calculate the contrasts. As shown in Fig. S2, the intensity cross sections of element set with d = 55 $\mu m$ and d = 62 $\mu m$ are shown. From these two plots, the average contrast ratio can be found as 18.10% and 32.28% respectively. As a result, we can claim that the resolution is 62 $\mu m$.

Here we show some numerical simulation results to estimate the theoretical resolution limit of this technique by varying different parameters. The simulation is based on the theory shown in the first section. As mentioned in the main paper, we investigate the influences of three different parameters: the thickness of detection crystal, the pixel size and the central wavelength of the THz pulse.

Three different thicknesses of detection ZnTe crystal are chosen with a 32 $\mu m$ pixel size and 128 pixels: 50 $\mu m$, 100 $\mu m$ and 200 $\mu m$. The central wavelength is 940 $\mu m$. As shown in Fig. S2(a)-(c), the reconstructed field with 50 $\mu m$ crystal thickness is the most clear one. By calculating the contrast of each element using the same method as mentioned in the main draft (the example

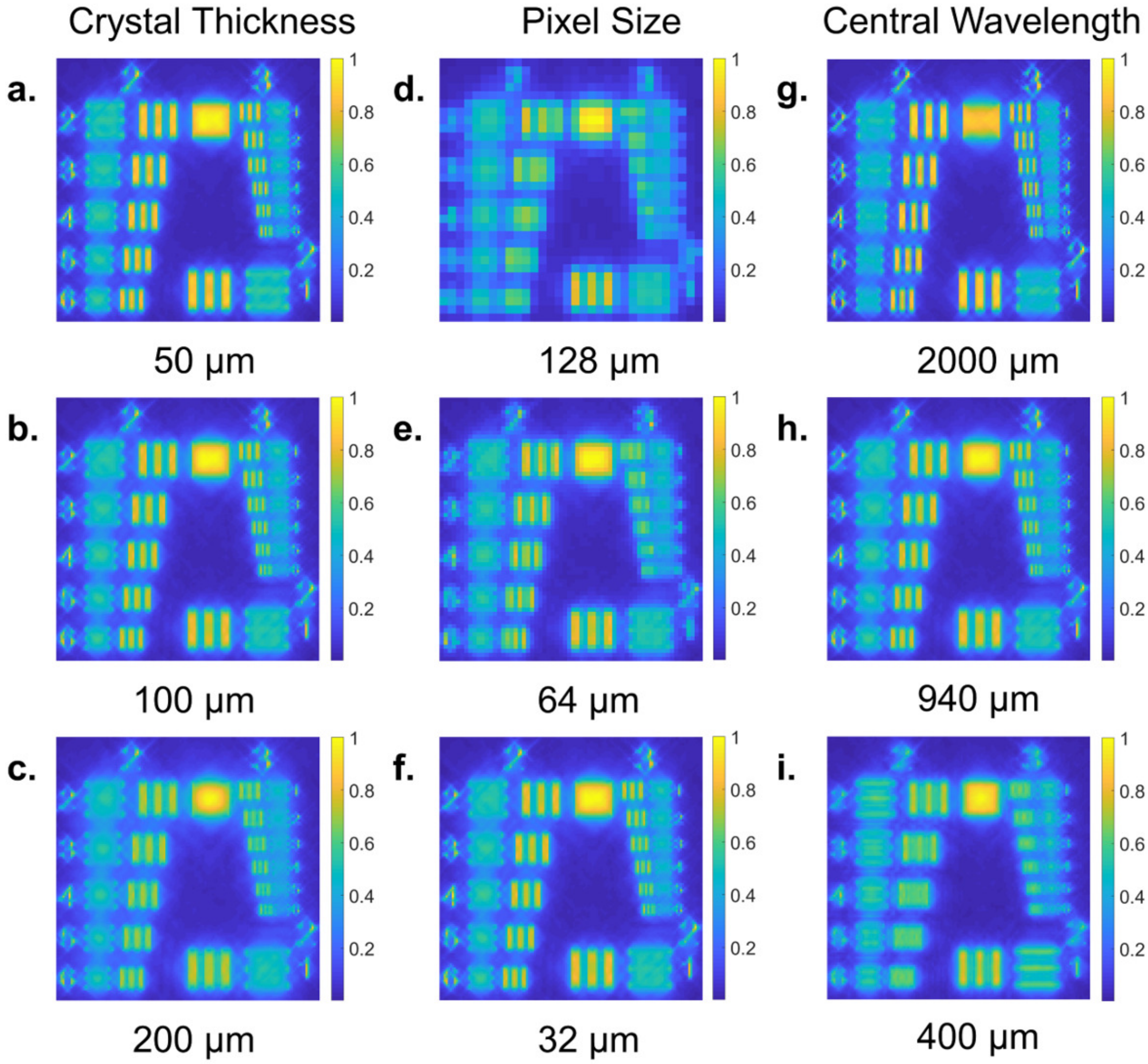


Fig. S2: (a) to (c). Normalized results with different crystal thicknesses. (d) to (f). Normalized results with different pixel sizes. (g) to (i). Normalized results with different central wavelengths.

of the cross sections can be found in Fig. S3(c)), we find that with 50 $\mu m$ and 100 $\mu m$ detection crystal thicknesses, even the element 3-6 (d = 35 $\mu m$) can be fully resolved with a 35.36% contrast and a 28.47% contrast respectively. As a comparison, the resolution in Fig. S2(c) is only 88 $\mu m$ with a 20.79% contrast. To estimate the resolution limit of our current setup, we choose a 20 $\mu m$ thick detection crystal with a 8 $\mu m$ pixel size. As shown in Fig. S3(d), under this configuration, the resolution can easily reach 8.8 $\mu m$ with a 30.00% average contrast. The reason why we change the pixel size is that, from the comparison below, a large pixel size can strongly limit the resolution even though the thickness is small. To reduce the pixelization effect, we change the pixel size here

to a lower level for a fair comparison. Therefore, a thin detection crystal can significantly increase the resolution.

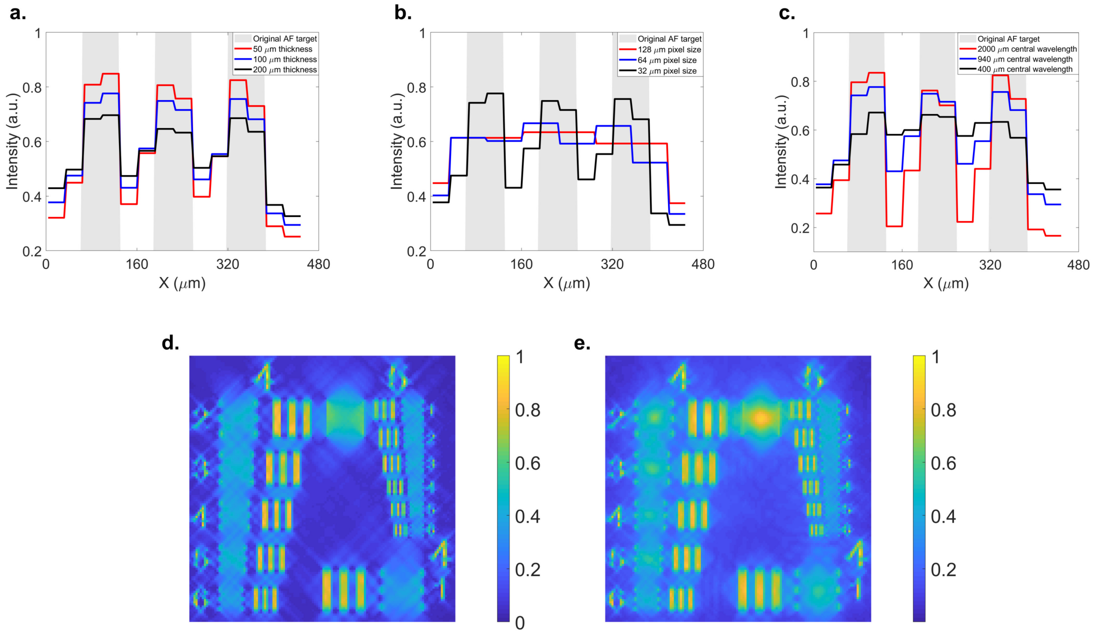


Fig. S3: (a). The intensity cross section comparisons with different crystal thickness. (b). The intensity cross section comparisons with different pixel size. (c). The intensity cross section comparisons with different central wavelength. The gray stripes stands for the intensity cross section of element 3-1 in the original AF target. (d). Recovered image with 20 $\mu m$ thick detection crystal and 8 $\mu m$ pixel size. (e). Recovered image with 100 $\mu m$ thick detection crystal and 8 $\mu m$ pixel size.

Three different pixel sizes are simulated under a 100 $\mu m$ thick detection crystal with a 940 $\mu m$ central wavelength THz pulse: 128 $\mu m$, 64 $\mu m$ and 32 $\mu m$. Since our field size is fixed, the number of pixels in each case then becomes 32, 64 and 128 respectively. The recovered figures are shown in Fig. S2(d)-(f) respectively. Through calculating the contrast using cross sections (as shown in Fig. S3(b)), the resolution in Fig. S2(d) is 125 $\mu m$ and the contrast is only 22.08%. As the comparison, the resolution in Fig. S2(e) is 88 $\mu m$ with a 25.96% contrast while the resolution in Fig. S2(f) is less than 35 $\mu m$ as we discussed in previous paragraph. These results shows that the pixelization effect can significantly reduce the resolution and contrast, and one can expect a better resolution if we use a smaller pixel size. Therefore, as shown in Fig. S3(e), when we decrease the pixel size to 8 $\mu m$, the resolution is found to be 11 $\mu m$ ($\lambda/86$) with a 33.7% average contrast.

That is to say, optical SLMs are more favorable than THz SLMs in computational imaging since the pixel size is much smaller, which indicates that our method can provide better performance than those methods with THz SLMs. One should note that since the pixel size cannot be infinitely small, the resolution in our approach will be limited by the pixel size projected on the detection crystal, which is a common resolution limit of computational imaging methods.

Three different central wavelength with a same spectrum shape are analyzed with a 100 $\mu m$ thick detection crystal: 2000 $\mu m$, 940 $\mu m$, 400 $\mu m$. The number of pixels is 128 so that the pixel size is 32 $\mu m$. Note that even the central wavelength has shifted, the shapes of the spectrum are same for all three cases. Intuitively speaking, Fig. S2(g) (2000 $\mu m$ central wavelength) has the best contrast while Fig. S2(i) is the worst. By calculating the contrast of the elements in all three figures (the example of the cross sections can be found in Fig. S3(c)), we find that the resolution in Fig. S2(i) is only 125 $\mu m$ with a 21.05%. That is to say, in the sub-wavelength region, the resolution in Fig. S2(i) is worse than the resolution in Fig. S2(c) even the detection crystal is much thinner. However, the resolution in Fig. S2(g) should be much less than 35 $\mu m$ since the contrast of element 3-6 is found to be 48.11%, which is much higher than the contrast of the same element in Fig. S2(a). Therefore, in the near-field region, a longer central wavelength pulse will provide a better resolution even when the detection crystal is thicker. The conclusion here is very different from the conclusion in far-field imaging where a shorter wavelength is always desired for a higher resolution.

## V. IMPROVED RESOLUTION COMPARED TO NEAR-FIELD EO IMAGING

The conventional near-field EO imaging utilizes the similar detection method as ours [8, 9]. One can use an optical CCD array to retrieve real time images of the THz field. Unlike other near-field imaging techniques, this EO imaging technique also provides noninvasive measurements with a concise and reliable setup. However, the resolution of this technique is not as good as our approach especially when the crystal goes thicker. The resolution limitations in both cases highly depend on the thickness of the detection crystal. However, this factor has less impact in our case because we measure the total electrical field of each spatial pattern as shown in Eqn. (3), which is an accumulation result through the whole thickness of detection crystal. Therefore, we can find a position $z'$ where the product of THz field, the $i_{th}$ spatial pattern and the thickness of the crystal $d$ is exactly equivalent to the integral in Eqn. (3). That is to say, we can rewrite the Eqn. (3) into

another form:

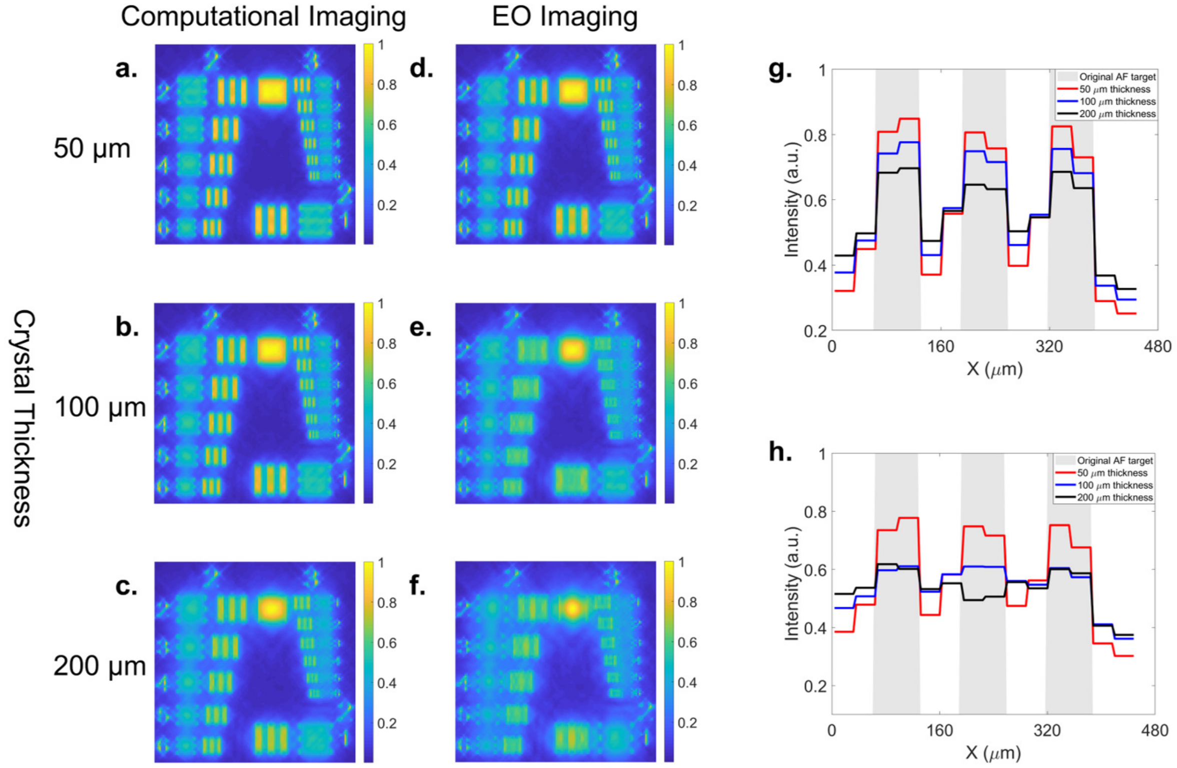


Fig. S4: (a) to (c). Normalized results using computational imaging methods. (d) to (f). Normalized results using EO imaging methods. The figure (a) and (d) both have a 50 $\mu m$ thick detection crystal while the crystal thicknesses in figure (b) and (e) are both 100 $\mu m$. The crystal thicknesses in figure (c) and (f) are 200 $\mu m$. (g) and (h). Intensity cross sections of element 3-1 using computational imaging and EO imaging respectively.

$$
\begin{aligned}
P_{\mathrm{s},i}(t) = & \int\int\int_{z_0}^{z_1} \frac{1}{c} I_i(x, y, z, \omega_1)\omega_1 L n_0^3(\omega_1) r_{14}(x, y)\times \\
& E_{\mathrm{THz}}(x, y, z, \omega_{\mathrm{THz}}, t) dxdydz \\
= & \int\int \frac{1}{c} z' I_i(x, y, z', \omega_1)\omega_1 L n_0^3(\omega_1) r_{14}(x, y)\times \\
& E_{\mathrm{THz}}(x, y, z', \omega_{\mathrm{THz}}, t) dxdy,
\end{aligned}
\tag{9}
$$

where $E_{\mathrm{THz}}(x, y, z', \omega_{\mathrm{THz}})$ and $I_i(x, y, z', \omega_1)$ are THz field and $i_{th}$ spatial pattern at distance $z'$ respectively. Note that $z'$ is less than $z_1$ so that the THz field at $z'$ is less diffracted than the field at $z_1$. Therefore, what the computational imaging recovers is not the field at the end of detection

crystal but the field at position $z'$. However, in the EO near-field imaging case, what the camera measures is the transverse structure of the THz field at the rear surface of detection crystal (i.e., $z_1$), which is more blurred due to the relatively stronger diffraction. In another word, when the detection crystal has a thickness $d = z_1 - z_0$, the field recovered by the computational imaging method with this crystal is equivalent to the field imaged by EO imaging with a thinner crystal thickness $d' = z' - z_0$. This difference in the measurement favors the superior resolution in our sampling technique by sacrificing the image acquisition time. Another drawback in the near-field EO imaging is the requirement of high power lasers [10]. As what we discuss in the last section, a thinner crystal can give better resolution but worse contrast and SNR. Therefore, to get high resolution images with a good contrast, a high energy laser is used to provide a strong THz field. However, in our case, high contrast images can be retrieved with a low intensity pump laser and a extremely weak probe.

To intuitively show the comparison, we compare the reconstructed images from computational imaging and the images from near-field EO imaging with different thicknesses of the crystal. As shown in Fig. S4(a)-(c), three different images with thicknesses 50 $\mu m$, 100 $\mu m$ and 200 $\mu m$ are reconstructed. To keep the comparison fair, this set of images all have 128 pixels with 32 $\mu m$ pixel size. After calculating the contrast of each element from intensity cross sections (as shown in Fig. S4(g) and (h)), the element 3-6 (d = 35 $\mu m$) can still be fully resolved in Fig. S4(d) but with a 26.11% contrast, which is much lower than the contrast using computational imaging method in Fig. S4(a). For Fig. S4(e) and (f), there is no element can be resolved. That is to say, our computational imaging method can significantly improve the resolution especially when the detection crystal is quite thick, which matches our prediction. Therefore, for applications requiring a thick detection crystal, our approach can provide much better performance than the conventional EO imaging. Therefore, under the same conditions, our computational method is superior than the conventional EO imaging in resolution and contrast with a sacrifice of imaging speed.


[1] Y.-S. Lee, *Principles of terahertz science and technology*, Vol. 170 (Springer Science & Business Media, 2009).

[2] X.-C. Zhang and J. Xu, *Introduction to THz wave photonics*, Vol. 29 (Springer, 2010).

[3] M. Harwit, *Hadamard transform optics* (Elsevier, 2012).

[4] J. Romberg, IEEE Signal Processing Magazine **25**, 14 (2008).

[5] E. Candes and J. Romberg, Inverse problems **23**, 969 (2007).

[6] C. Li, *Compressive sensing for 3D data processing tasks: applications, models and algorithms*, Ph.D. thesis, Rice University (2011).

[7] K. Murate, M. J. Roshtkhari, X. Ropagnol, and F. Blanchard, Optics letters **43**, 2090 (2018).

[8] Q. Chen, Z. Jiang, and X.-C. Zhang, in *Conference on Lasers and Electro-Optics* (Optical Society of America, 2000) p. CThR3.

[9] X. Wang, Y. Cui, W. Sun, J. Ye, and Y. Zhang, Optics Communications **283**, 4626 (2010).

[10] D. M. Mittleman, Optics express **26**, 9417 (2018).
